# Disparities in Coronavirus Disease 2019 Mortality among Japanese and Non-Japanese Residents: A Natural Experimental Study

**DOI:** 10.31662/jmaj.2025-0493

**Published:** 2026-06-05

**Authors:** Russell Miller, Kuniko Arita, Aya Yumino, Akira Shibanuma, Yoshimasa Kawazoe, Masamine Jimba

**Affiliations:** 1 Department of Community and Global Health, Graduate School of Medicine, The University of Tokyo, Tokyo, Japan; 2 Find a Doc, Japan (NPO), Toyama, Japan; 3 Department of International Cooperation for Medical Education, Graduate School of Medicine, The University of Tokyo, Tokyo, Japan; 4 Yokohama City Kotobuki-cho Health and Welfare Clinic, Yokohama, Japan; 5 Department of Biomedical Informatics, Graduate School of Medicine, The University of Tokyo, Tokyo, Japan; 6 Department of Environmental and Sustainable Engineering, Chulalongkorn University, Bangkok, Thailand

**Keywords:** epidemiology, Japan, COVID-19, equity, migrant, natural experiment

## Abstract

**Introduction:**

Universal health coverage has been cited as a critical factor in Japan’s success in limiting mortality due to coronavirus disease 2019 (COVID-19). It is unclear if this success extended to non-Japanese residents, especially prior to roll out of COVID vaccines. Non-Japanese residents represent a growing minority of Japan’s population, so this study aimed to assess any disparities in COVID-19 mortality between Japanese nationals and non-Japanese residents.

**Methods:**

In this natural experiment, mortality due to COVID-19 was derived from death certificate data (2020-2021) registered by the Ministry of Health, Labour and Welfare (MHLW). Mortality rates were compared between Japanese nationals and non-Japanese residents, and then mortality was further explored by nationality. (South) Korean residents were identified as a subgroup of interest due to their status as the most populous non-Japanese nationality historically and their disproportionately high mortality rates. Age-standardized mortality rates and years of potential life lost (YPLL) were computed using vital statistics and life expectancy data from MHLW.

**Results:**

Of the 20,775 COVID-19 deaths in 2020/21, 98.3% were Japanese nationals, while non-Japanese residents accounted for the remaining 358 deaths. The nationwide age-standardized mortality rate was 16.5 per 100,000 individuals, with 14.9 for non-Japanese residents. Among older adults (≥65 years), the mortality rate for non-Japanese residents was 152.3 per 100,000, compared to 52.5 for the general population. Koreans accounted for 193 (53.9%) of non-Japanese deaths. Crude mortality rate was 51.5 per 100,000 for this group, sharply increasing among older adults (162.1). Total YPLL due to COVID-19 in 2020/21 was 76,544.1 years, with YPLL rates of 59.5 years per 100,000 among Japanese nationals, 178.4 for non-Japanese residents, and 268.2 for Koreans.

**Conclusions:**

Notable disparities in COVID-19 mortality were uncovered, particularly among older non-Japanese and Korean residents. With growing diversity domestically, these findings suggest a need for institutional monitoring of health outcomes by nationality to better inform equitable public health in Japan.

## Introduction

Japan’s Universal Health Coverage (UHC) system played a key role in mitigating the impact of the coronavirus disease 2019 (COVID-19) pandemic, but as the country’s demographic landscape evolves, more comprehensive big data analysis is necessary to assess health equity for all residents, especially the growing non-Japanese population ^(1)^. From 2015 to 2021, the number of international migrants in Japan (i.e., international residents in Japan without Japanese citizenship) surged by 45.6% to approximately 2.7 million (2.2% of Japan’s population) ^(2)^. Although Japan has made inclusion a national priority, most recently emphasized in Health Japan 21 (Third Term) ^(3)^, that envisions a sustainable society where all people can live healthy and fulfilling lives, current assessments of non-Japanese residents have often been fragmented, limiting the understanding of their healthcare needs. A more systematic approach using large-scale, integrated datasets that includes administrative records, electronic health records, insurance claims, and other real-world data sources ^(4)^ is essential for identifying disparities and guiding evidence-based interventions.

The COVID-19 pandemic, widely described as a natural experiment, was an unplanned, large-scale disruption that exposed underlying disparities in health systems and outcomes ^(5), (6)^. This framing has been used to evaluate the effects of lockdowns ^(7)^, healthcare disruptions ^(8)^, and vaccination policies ^(9)^ across countries. Studies assessing this natural experiment have revealed disparities in infection and mortality risks by socioeconomic status, occupation, and ethnicity ^(10)^. Such an unprecedented situation provided insights into how existing systemic inequities could amplify the effects of a sudden public health crisis in terms of excess mortality ^(11)^, particularly among vulnerable groups.

Between 2020 and 2021, the average global mortality rate due to COVID-19 was approximately 66 deaths per 100,000 people, whereas in Japan, it was much lower, at around 14 deaths per 100,000 people ^(12)^. In the United States (US), with a private insurance market, the mortality rate was about 230 deaths per 100,000 people. In other high-income countries with UHC, like Italy and Norway, the mortality rates were around 221 and 24 deaths per 100,000 people, respectively. These differences suggest that though universal healthcare can be beneficial, other factors, such as public health policies, vaccination strategies ^(13)^, and social determinants of health, also play a crucial role in impacting COVID-19 mortality across countries.

Non-Japanese residents may face unique challenges in accessing healthcare services, such as language barriers, limited knowledge of available resources, or systemic inequities in healthcare provision ^(14)^. Unlike other high-income countries, few publications are available that compare the health of minorities to the that of majority population in Japan. In terms of COVID-19 infections, there is only one epidemiological study assessing clinical outcomes in Japanese nationals and non-Japanese residents. Using the COVID-19 infection registry, the researchers performed propensity score matching based on sex, age, body mass index (>25 kg/m^2^), and several comorbidities ^(15)^. No significant differences in health outcomes were found, and all outcomes were descriptively better for non-Japanese residents, including lower rates of oxygen use, non-fatal complications, and death.

Although previous research has shown that all-cause mortality outcomes between Japanese nationals and non-Japanese residents were largely equitable during the pandemic ^(16)^, disparities in access to healthcare services persisted. Notably, non-Japanese residents reported difficulty in finding medical facilities ^(17)^, and relied heavily on informal information sources (e.g., social media) ^(17)^; news reports indicated that up to 20% fewer non-Japanese residents were vaccinated compared to Japanese nationals in 2021 ^(18)^.

Rather than all-cause mortality, this study aimed to investigate disparities in mortality due to COVID-19 between Japanese nationals and non-Japanese residents in Japan. By examining these differences at various administrative levels, the study provides insights on the protection UHC may offer for diverse populations and identifies areas where healthcare access can be improved to ensure equitable outcomes for all residents.

## Materials and Methods

### Study design

This observational study employed a natural experimental design, analyzing real-world data collected during the early period of COVID-19 pandemic from 2020 to 2021. The study design took advantage of the naturally occurring variation in COVID-19 mortality across different nationalities residing in Japan, comparing Japanese nationals and non-Japanese residents. A key contextual factor was Japan’s border policy: from April 3 to August 31, 2020, non-Japanese―including legal residents―were denied re-entry into the country ^(19)^. Despite this, the non-Japanese resident population increased by approximately 1% between 2018 and 2021, while the Japanese population decreased by about 1% ^(20)^. Over this entire period (January 2018-December 2021), the Japanese population decreased by 0.9 million people (about 1.2% of the country’s total population), while the migrant population on “residential visa” (i.e., not tourist visa) increased from 2.73 million in 2018 to 2.93 million in 2019, before declining to 2.76 million in 2021 (overall increase of about 1.1% over the entire study period). The proportion of international migrants in Japan remained consistent at roughly 2.2% of the national population over this period. This setting allowed for descriptive analysis of differences in outcomes between groups without intervention or randomization, consistent with a natural experiment approach in a real-world context.

### Data source

This study utilized national vital statistics microdata from Japan’s Ministry of Health, Labour and Welfare (MHLW) to assess COVID-19-related individual deaths by nationality from 2020 to 2021. The data includes all deaths registered in Japan, excluding individuals temporarily residing in Japan without a residency card (*zairyūkādo*), such as tourists. Only deaths occurring within Japan’s borders were considered. The dataset also captured demographic details, such as nationality, age, sex, and region of residence. COVID vaccination history or any clinical data, such as comorbidities, is not available as part of such national death records. Missing data were not imputed.

All mortality rates were calculated to the municipality level using population estimates from the 2020 Japan National Census for all nationalities ^(21)^. According to the death records, no children under 14 years of age died of COVID-19; therefore, only working-age populations (15-64 years and above) were included from the census. Notably, 2,202,484 individuals who did not report nationality in the 2020 Census were excluded from analyses by nationality. Only nationalities reported by MHLW could be disaggregated, including a group consisting exclusively of South Korean nationals (hereafter “Koreans”). Following Centers for Disease Control National Vital Statistics guidance, mortality rates based on fewer than 20 deaths were not reported because rates derived from such small counts have high relative standard error and are considered statistically unreliable ^(22)^.

### Analysis

COVID-19-related deaths were identified using the International Classification of Diseases, 10th Revision code U07. Mortality trends were disaggregated by nationality, comparing Japanese nationals with non-Japanese residents. The study also explored disparities in mortality by age, sex, and nationality, emphasizing the potentially different health outcomes for Korean residents, who represented the largest non-Japanese resident group in Japan and exhibited particularly high mortality. To assess the potential impact of deaths among Koreans on the non-Japanese population, further sensitivity analyses were conducted using the non-Japanese residents, excluding Koreans.

National life expectancy data from 2019, provided by MHLW (https://www.e-stat.go.jp/dbview?sid=0003411893), were used to calculate sex-standardized years of potential life lost (YPLLs) due to premature mortality for Japanese nationals and non-Japanese residents. Alternative reference life tables (Korea) were examined in sensitivity analyses ^(23)^; primary results are based on the Japanese life table. Due to data limitations, YPLL was approximated using the average age at death rather than with age-specific death counts; this approach may underestimate YPLL, particularly in populations with wider death age distributions.

Descriptive statistics were calculated for COVID-19 mortality rates, age standardized rates (using age groupings from the 2020 census), and YPLL allowing for comparisons among different nationalities and regions. All statistical calculations were performed with either Excel for Windows or R version 4.2.2 (R Core Team, 2022).

## Results

### Overview of COVID-19 mortality trends

**Figure 1.**
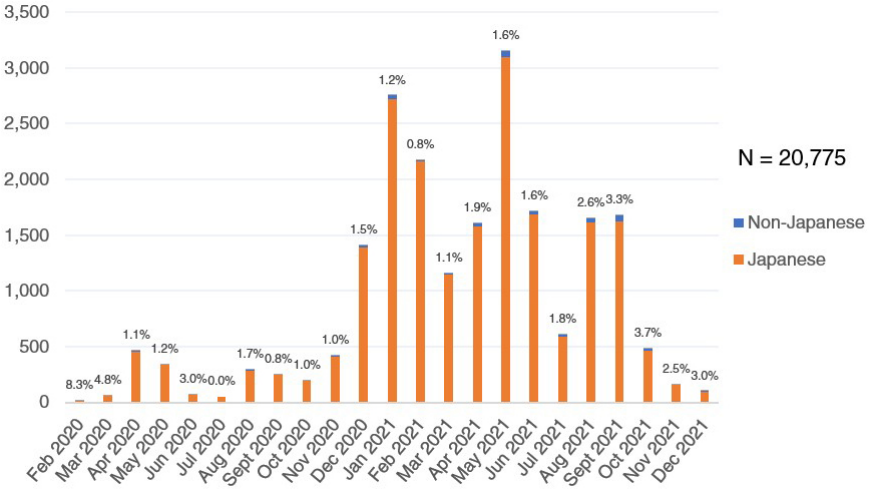
Trends in mortality due to COVID-19 from 2020 through 2021 (Infection Waves 1 through 5), including proportion of deaths among non-Japanese residents. COVID-19: coronavirus disease 2019.

A total of 20,775 deaths due to COVID-19 occurred between February 2020 and December 2021, with 1.7% (358 deaths) among non-Japanese residents and 98.3% (20,417 deaths) among Japanese nationals. The youngest recorded death was at 15 years old and the oldest was at 107 years old. Figure 1 illustrates the percentage of deaths due to COVID-19 among Japanese nationals and non-Japanese residents. This figure represents deaths recorded from the first COVID-19 death in February 2020 to December 2021, covering five distinct waves of the pandemic. The number of COVID-19 deaths was initially low, with 12 deaths in February 2020, and it increased to a peak of 3,148 deaths in May 2021. Notable peaks occurred in January 2021 (2,751 deaths) and May 2021 (3,148 deaths), with smaller peaks in February 2021 (2,174 deaths) and August 2021 (1,652 deaths). Death counts were lower in other months, such as July 2020 (49 deaths) and December 2021 (95 deaths).

### COVID-19 mortality by age group and nationality

Table 1 presents the cumulative mortality by age group and nationality. The overall median age at death was 82 years, with a median age of 83 years for Japanese nationals and 74 years for non-Japanese residents. Among individuals aged 15-64 years, 10.4% of Japanese deaths and 30.4% of non-Japanese deaths occurred in this age group. In contrast, 89.6% of deaths among Japanese nationals occurred in individuals aged 65 years and older, compared to 69.6% of deaths among non-Japanese residents.

**Table 1.** Demographics of Deaths and Mortality Rates Due to COVID-19 in Japan.

	Overall		Japanese		Non-Japanese	
All ages (n [%)])	20,775	100	20,417	98.3	358	1.7
Mortality rate	16.5		16.8		14.9	
Age group: 15-64 years (n [%])	2,223	10.7	2,114	10.4	109	30.4
Mortality rate	3.0		3.0		5.5	
Age group: 65 years and older* (n [%])	18,552	89.3	18,303	89.6	249	69.6
Mortality rate	52.5		52.1		152.3	
Male (n [%])	1,2191	58.7	11,981	58.7	210	58.7
Median age (years; IQR)	82	15	83	14	74	22

Age-standardized mortality rates. Age strata with largest proportion of deaths for all non-Japanese nationality groups. Age group percentages add up to 100% of “all ages”. COVID-19: coronavirus disease 2019; IQR: interquartile range.

### Mortality rates by nationality

Table 1 also describes the mortality rate based on population by nationality. Among Japanese nationals, age-standardized mortality rate was 16.8 per 100,000 individuals. The large majority of these deaths occurred among those aged 65 years and older, with an age-standardized mortality rate of 52.5 per 100,000 individuals. Among non-Japanese residents, there were 358 deaths, corresponding to an age-standardized mortality rate of 14.9 per 100,000 individuals. For non-Japanese residents aged 65 years and older, the age-standardized mortality rate was 152.3 per 100,000 individuals. There was little variation in sex distribution between nationality groupings.

Table 2 provides mortality data for specific nationalities within the non-Japanese resident group. Korean residents accounted for the largest number of deaths (193), with a crude mortality rate of 51.5 per 100,000 individuals. Other nationalities, such as Chinese, Filipino, and American residents, had lower crude mortality rates, ranging from 6.0 to 12.5 per 100,000 individuals. Mortality rates for Brazilian and Peruvian residents were relatively higher, at 17.2 and 17.1 per 100,000 individuals, respectively. When Koreans were removed from the analysis of non-Japanese residents, crude mortality rates dropped from 13.0 to 8.1. For individuals over 65 years of age, age-standardized mortality rates dropped from 152.3 to 134.8 when the Korean population was excluded.

**Table 2.** Demographics of Deaths and Mortality Rates among Non-Japanese Residents Due to COVID-19 in Japan by Country of Origin.

	Non-Japanese*		Non-Japanese without Koreans		Korea		China		Brazil		Philippines		Peru**		USA**		Thailand**		England**		Others	
All ages (n [%])	358	100	165	46.1	193	53.9	40	11.2	31	8.7	26	7.3	7	2.0	6	1.7	3	0.8	1	0.3	51	14.2
Mortality rate	14.9		8.1		51.5		6.0		17.2		11.3		17.1		12.5		7.0		7.4		6.3	
Age group: 15-64 years (n [%])	109	30.4	86	52.1	23	6.4	11	27.5	17	54.8	19	73.1	NC	NC	NC	NC	NC	NC	NC	NC	30	58.8
Mortality rate	5.5		4.9		8.5		NC		NC		NC		NC		NC		NC		NC		4.3	
Age group: 65 years and older* (n [%])	249	69.6	79	47.9	170	88.1	29	72.5	14	45.2	7	26.9	NC	NC	4	NC	NC	NC	NC	NC	21	41.2
Mortality rate	152.3		134.8		162.1		126.2		NC		NC		NC		NC		NC		NC		140.0	
Male (n [%])	210	58.7	106	64.2	104	53.9	22	55.0	23	74.2	13	50.0	NC	NC	NC	NC	NC	NC	NC	100	35	49.0
Median age (years; IQR)	74	22	63.0	21	81	16	73.5	20.5	64.0	14.0	58.5	17.5	NC	NC	NC	NC	NC	NC	NC	NC	61	19.5

Korean data label refers to individuals with South Korean nationality throughout the analysis. COVID-19: coronavirus disease 2019; IQR: interquartile range; NC: not calculated due to deceased populations with >20 individuals; USA: United States of America. *Age-standardized mortality rates. Others are crude mortality rates calculated using population data from the 2020 Japan Census, unless otherwise noted. **Precision of these mortality rates should be treated with caution due to the population being <20 individuals.

### Mortality rates by administrative region and nationality

The cities of Osaka (n = 79), Kobe (n = 57), and Tokyo (n = 56) had the largest total number of deaths among non-Japanese residents. Table 3 provides the mortality rates of COVID-19 per 100,000 individuals, disaggregated by administrative region, nationality, and age group. More specifically, Osaka’s Ikuno Ward showed particularly high mortality rates, with 93.5 overall and 105.7 among non-Japanese residents. Japanese nationals had a mortality rate of 99.0, whereas Korean residents had a mortality rate of 132.9 overall, increasing to 309.6 among those aged 65 years and older.

**Table 3.** Outlier Municipalities in Terms of Non-Japanese Mortality Due to COVID-19.

Municipalities*	Proportion of non-Japanese among local population	Mortality rate (per 100,000 individuals)							
		Overall		Japanese		Non-Japanese		Korean	
		Mortality rate	65 years and over	Mortality rate	65 years and over	Mortality rate	65 years and over	Mortality rate	65 years and over
Ikuno Ward, Osaka City, Osaka Prefecture	18.6%	93.5	295.6	99.0	291.8	105.7	313.6	132.9	309.6
Nagata Ward, Kobe City, Hyogo Prefecture	5.2%	122.4	359.5	115.9	315.6	406.8	1109.2	609.0	1220.3

COVID-19: coronavirus disease 2019. *The only municipalities with 20 or more non-Japanese deaths, all deaths except one were among South Koreans in these municipalities.

Kobe’s Nagata Ward had the highest overall mortality rates, with 122.4 per 100,000 individuals and 406.8 among non-Japanese residents. The mortality rate among Japanese nationals was 115.9, and Korean residents experienced a significant mortality rate of 609.0, which rose further to 1,220.3 for those aged 65 years and older.

### Approximate YPLL due to COVID-19 mortality

Table 4 provides an overview of the YPLL due to COVID-19 mortality. Overall, an estimated 76,544.1 YPLL between 2020 and 2021 were due to COVID-19. Disaggregated by nationality, total YPLL was 72,258.4 among Japanese nationals, with 59.5 YPLL per 100,000 individuals. In contrast, the non-Japanese population experienced 4,285.7 YPLL, corresponding to 178.4 YPLL per 100,000 individuals. Korean residents experienced particularly high numbers, with 1,004.7 total YPLL and 268.2 YPLL per 100,000 individuals. When recalculated using life expectancy from Korea, Korean residents in Japan had lower YPLL: 786.9 total and 210.1 per 100,000 individuals. Non-Japanese individuals, excluding Koreans, experienced 161.8 YPLL per 100,000 individuals, which was even lower than the 178.4 YPLL among all non-Japanese residents.

**Table 4.** Expected Years of Potential Life Lost by Nationality Group.

	Total years of life lost	Years of life lost per 100,000 individuals
Overall	76,544.1	61.8
Japanese	72,258.4	59.5
Non-Japanese	4,285.7	178.4
Korean	1,004.7	268.2
Koreans using Korean life expectancy	786.9	210.1
Non-Japanese without Koreans	3,281.0	161.8

Life expectancy for non-Japanese residents assumed to be the same as Japanese nationals.

## Discussion

This study reveals considerable disparities in COVID-19 mortality between Japanese nationals and non-Japanese residents, suggesting that while effective in reducing overall mortality, the public health response did not ensure equitable outcomes across all populations. Our findings show that overall deaths varied over time, with peaks in January and May 2021. Notably, the proportion of COVID-19 deaths among non-Japanese residents exceeded 3% beginning in September 2021, despite representing a smaller share of the overall population. This increase (occurring after the fifth wave) may indicate cumulative exposure risks and other structural factors prior to the introduction of COVID vaccines in Japan. Additionally, non-Japanese residents tended to be younger at death, resulting in a higher burden of YPLL. Although the overall age-standardized mortality rate was lower among non-Japanese residents, certain groups, especially Korean residents, experienced higher rates. Finally, regional analysis revealed marked differences in COVID-19 mortality rates, implying that local factors may be contributing to these disparities, and warrant further investigation.

The higher mortality rates and YPLL observed among non-Japanese residents, particularly Korean residents, suggest that there are critical gaps in healthcare access and utilization for non-Japanese residents. A study analyzing COVID-19 mortality trends in Japan found that non-Japanese patients were more likely to be younger and engaged in occupations with higher exposure risks, such as the service industry ^(15)^. Despite these increased risks, the researchers did not find significant disparities in clinical outcomes between Japanese and non-Japanese patients after adjusting for factors like age and comorbidities. However, the researchers emphasized the need for further investigations into the social determinants of health affecting non-Japanese populations. These disparities highlight the need for targeted policies that address the unique challenges faced by non-Japanese residents, such as language barriers, cultural differences, limitations in accessing healthcare services, and potential discrimination.

Vaccine uptake is one possible confounder in such COVID-19 analyses; however, vaccinations were not available in Japan for almost entire study period in question. Vaccinations began for those aged 65 years and older in April 2021 in Japan and vaccination opened to all residents aged 18 years or older from June 2021. Vaccination coverage disparities were evident with non-Japanese residents having significantly lower second dose coverage compared to that of the general population ^(24)^ in the few municipalities that made this information public, highlighting barriers in vaccine access and uptake among non-Japanese residents ^(18)^.

Research has concluded that all-cause mortality between Japanese nationals and non-Japanese residents was not exacerbated by the pandemic, suggesting that COVID-19 did not worsen general health inequities in this context ^(16)^. However, the pandemic serves as a natural experiment where disparities based on social determinants of health may be revealing themselves in real-time, particularly in terms of healthcare access and uptake among non-Japanese residents. The study of the COVID-19 period identified an acculturation effect among older adults, where initially lower mortality rates for non-Japanese residents (healthy migrant effect) converged with those of the majority population as migrants aged ^(16)^. However, though this trend was true in aggregate, it did not hold when broken down by age group.

Notably, Koreans were the only nationality with a higher crude mortality rate than Japanese nationals. Further age-standardized analysis revealed that older Koreans had significantly higher mortality rates compared to that of older Japanese nationals. Approximately 55% of non-Japanese residents in Japan during this period were aged 20-39 years, whereas nearly a third of Japanese nationals were aged 65 years or older ^(25)^, leading to a skew in crude analysis. Due to the small number of deaths among non-Korean non-Japanese nationalities, age-standardized rates were not calculated for these groups. Research on similar mortality data from MHLW up to 2006 demonstrates similar trends in standardized mortality rates between ethnic Koreans and Japanese nationals in Japan, with lower rates for Koreans ^(26), (27)^.

The observed regional differences in COVID-19 mortality rates highlight potential disparities in exposure risk, healthcare access, and socioeconomic conditions among non-Japanese residents. Kobe’s Nagata Ward and Osaka’s Ikuno Ward, both known for their large, multi-generational (*zainichi*) Korean communities who have a separate identity from Japanese nationals ^(28), (29), (30)^, exhibited notably high mortality rates, particularly among older Korean residents. For older Korean residents in particular, prior studies have documented their disproportionately high reliance on public assistance and long-standing experiences of discrimination ^(29)^. Qualitative reports suggest that some Korean welfare recipients faced stigma in healthcare settings―sometimes being told directly that they were a burden to society. This dual discrimination, both as ethnic minorities and as welfare recipients, may discourage timely care-seeking and may contribute to poorer outcomes. Though peer-reviewed studies on this phenomenon remain limited, practitioner accounts have begun to document such incidents ^(31)^.

These findings align with previous studies suggesting that ethnic minority populations often experience a disproportionate burden of infectious diseases due to structural inequities, including crowded living conditions, lack of pension ^(32)^, and barriers to healthcare access ^(33), (34)^. In the US, for example, during the initial waves of the COVID-19 pandemic, non-Hispanic Black adults had a COVID-19 death rate 3.4 times higher than that of non-Hispanic White adults ^(35)^.

The strikingly high mortality rates among older Korean residents in Kobe and Osaka, exceeding 1,200 deaths per 100,000 individuals in one case, warrant further investigation into potential social and healthcare disparities that may have exacerbated their vulnerability. In contrast, other research has determined that YPLL due to COVID-19 in South Korea itself was the lowest among the 20 countries examined ^(36)^. Addressing these inequities through targeted public health strategies, improved outreach, and culturally competent healthcare services could help mitigate such disparities in future health crises ^(37)^. In order to utilize more big health data streams as part of machine learning algorithms to monitor population health, it is critical that nationality and/or ethnicity data are collected as part of standard of care in Japan.

The overwhelming majority of municipalities had comparatively low COVID-19 mortality rates despite their large non-Japanese populations. This contrast may reflect more effective local public health engagement or differing community structures. For example, Hamamatsu’s Chuo Ward is home to a large Brazilian community rather than Korean, supported by dedicated services including a Brazilian consulate and a school system, which may have facilitated better communication and healthcare access ^(38)^. Similarly, a mixed methods study of Nepali residents pointed to a lack of knowledge about Japan’s healthcare system as a key reason about not seeking treatment for COVID-19 ^(8)^. Lower mortality rates in some urban areas with large non-Japanese populations suggest that local factors, such as differences in community transmission dynamics, trust in public health information, or healthcare engagement, may influence outcomes.

The findings emphasize that Japan’s UHC system may not be fully inclusive in practice. Despite the coverage being available to all residents, systemic barriers may prevent non-Japanese residents from benefiting equally. Factors such as difficulty in navigating healthcare systems, lack of culturally appropriate information, and socioeconomic disparities (discrimination against welfare recipients compounded by discrimination against multigenerational Koreans) likely contribute to the observed differences in mortality outcomes. Public health interventions that focus on improving accessibility and on providing culturally tailored healthcare are necessary to bridge these gaps ^(39)^.

The findings of this study are consistent with previous research on health inequalities among migrant populations in other countries ^(10)^, indicating that equitable healthcare remains a global challenge. Finally, as the non-Japanese resident population in Japan ages, outreach programs that specifically address the needs of older non-Japanese residents may become more critical ^(40)^. Ensuring that this particular population receives timely information and support during health crises may be most crucial for reducing ethnic disparities in health outcomes.

### Strengths and limitations

Strengths of this study include its use of comprehensive national vital statistics microdata from Japan, which allowed for a detailed analysis of COVID-19 mortality disparities between Japanese nationals and non-Japanese residents. The study also employed standardized measures, such as YPLL and age-standardized mortality rates, which enhance comparability and reliability of the findings across diverse populations. By disaggregating data down to the municipal level, this study provides valuable insights into local disparities, and helps to identify specific regions and populations that may require targeted public health interventions.

However, this study also has several limitations. First, it is a retrospective observational study, which means it cannot establish causation but only identify associations. Second, there may be residual confounding factors that were not accounted for, such as differences in socioeconomic status, underlying health conditions, and access to healthcare facilities. Additionally, the use of census estimates for population data may introduce some inaccuracy in the mortality rate calculations, particularly for municipalities with rapidly changing population dynamics.

This study highlights significant disparities in COVID-19 mortality between Japanese nationals and non-Japanese residents in Japan. Although Japan’s UHC was effective in reducing overall COVID-19 mortality, non-Japanese residents, particularly older adults and Korean residents, experienced higher mortality rates and a greater burden of YPLL. These findings suggest that awareness and targeted interventions may be needed to address healthcare inequalities and to ensure equitable health outcomes for all residents, regardless of nationality. Improved data collection on non-Japanese residents and tailored healthcare policies could help mitigate the disparities observed in this study, and promote a more inclusive healthcare system.

## Article Information

### Author Contributions

Russell Miller and Masamine Jimba conceptualized the study. Russell Miller and Kuniko Arita acquired the secondary data and managed ethical review. Russell Miller analyzed the data with input from Masamine Jimba, Akira Shibanuma, Yoshimasa Kawazoe, and Aya Yumino. Russell Miller wrote the first draft of the manuscript. All authors read and approved the final manuscript.

### Conflicts of Interest

Russell Miller is an employee of Syneos Health Japan K.K. and is serving as President of Find a Doc, Japan (a registered NPO in Japan, https://www.findadoc.jp/). This research was conducted completely separate from any activities related to these employments.

### IRB Approval Code and Name of Institution

This study received ethical approval from the ethics committee of the Graduate School of Medicine, The University of Tokyo (ID: 2022055NI). This study exclusively involves secondary data analysis utilizing de-identified datasets. This study does not involve direct interaction with human subjects.

### Availability of Data and Materials

The raw data for this study is available from the Ministry of Health, Labour and Welfare upon application for microdata and is not publicly available for ethical reasons. However, aggregated data are available from the corresponding author on reasonable request.
